# Staged surgery for closed Lisfranc injury with dislocation

**DOI:** 10.3389/fsurg.2022.984669

**Published:** 2022-08-19

**Authors:** Wenbao He, Jiang Xia, Haichao Zhou, Zhendong Li, Youguang Zhao, Yunfeng Yang, Bing Li

**Affiliations:** Department of Orthopedics, Shanghai Tongji Hospital, School of Medicine, Tongji University, Shanghai, China

**Keywords:** staged surgery, lisfranc injury, surgical technique, treatment, surgical outcome

## Abstract

**Objective:**

To investigate the clinical efficacy of staged surgery for patients with closed Lisfranc injury and dislocation.

**Methods:**

This study included 48 patients with acute closed Lisfranc injury and dislocation admitted between July 2016 and July 2021. The patients were divided into two groups. 23 patients in group A underwent staged surgeries included emergency reduction within 4–8 h after injury, and open reduction and internal fixation of Lisfranc injury and first tarsometatarsal joint fusion after the swelling had subsided. 25 patients in group B underwent open reduction and internal fixation as an elective procedure after the swelling had subsided. American Orthopedic Foot and Ankle Society (AOFAS) midfoot scores and visual analog scale (VAS) scores were used for assessment at the final follow-up.

**Results:**

A total of 48 patients with closed Lisfranc injury and dislocation were included. The lengths of hospitalization were 11.52 ± 1.61 day and 19.80 ± 2.37 day in groups A and B, respectively. The total lengths of surgery were 67.34 ± 1.71 min and 104.36 ± 8.31 min in groups A and B, respectively. 48 patients completed the final follow-up (follow-up period range: 12–24 months, mean: 18 months). All fractures had healed at 12–18 weeks after surgery (mean: 14.6 weeks). At the 1-year postoperative follow-up, the AOFAS and VAS score was 86.87 ± 4.24 and 1.91 ± 0.78, respectively, during weight-bearing walking in group A patients and 71.72 ± 5.46 and 3.20 ± 1.17 in group B. By the end of the follow-up period, only 2 patients in group B had developed traumatic arthritis and no patients had joint re-dislocation or required secondary surgery.

**Conclusion:**

Staged surgery for closed Lisfranc injury with dislocation reduced the incidence of perioperative complications and achieved good surgical outcomes while shortening the lengths of surgery and hospitalization.

## Introduction

Acute Lisfranc injuries account for approximately 0.2% of all fractures and dislocations. Most are closed injuries, with high-energy injuries such as motor vehicle collisions and falls from height leading to severe dislocated Lisfranc injuries and accounting for approximately 40%–45% of injuries (1–3), Due to the severity of these injuries, surgical treatment is difficult, postoperative complication rates are high, and a second salvage joint fusion is usually required once traumatic arthritis develops (4), Therefore, a rational treatment plan is vital.

There remains debate over the best surgical treatment for high-energy Lisfranc injuries, as well as when and how to operate (3, 5, 6). The timing of surgery depends on soft tissue conditions, with minimal soft tissue swelling at 6–8 h and 7–14 day post-injury indicating the best time to operate. Lisfranc injuries with severe dislocation often develop blisters, which prolong the waiting time for surgery and may even lead to fascial compartment syndrome. Moreover, waiting for the swelling of the affected limb to subside before surgery can lead to intraoperative reduction difficulties, thus leading to poor treatment outcomes. However, emergency open reduction and internal fixation (ORIF), a procedure prone to postoperative incisional complications, is rarely used in clinical practice. How to balance the advantages and disadvantages of emergency surgery and elective surgery becomes the key to optimizing the treatment plan for Lisfranc injuries with dislocation.

Notably, for high-energy injury involving the joint at other sites, such as pilon or tibial plateau fractures, it has been proved that staged treatment can significantly improve patient outcomes (7–10). Therefore, inspired by the concept of staged treatment, this study of 48 patients with closed dislocated Lisfranc injuries who were admitted for trauma between July 2016 and July 2021 to investigate the clinical efficacy of staged surgery (emergency incisional decompression and temporary fixation, and elective strong internal fixation) for closed Lisfranc injuries with dislocation.

## Patients and methods

### Inclusion and exclusion criteria

The inclusion criteria were: (i) Closed Lisfranc injury with dislocation. (ii) Acute injury. (iii) Age ≥18 years.

The exclusion criteria were: (i) Old Lisfranc joint injury. (ii) Multiple injuries or pathological fractures. (iii) Foot deformity, variation, or history of foot surgery.

### General information

Dislocations of the tarsometatarsal joints were classified as three types (homolateral partial displacement, homolateral total displacement, divergent total displacement) with or without associated fractures. The patients were injured in car accidents or falls from height. All patients had undergone preoperative anteroposterior and oblique foot radiography and computed tomography (CT) plain three-dimensional (3D) reconstruction. The patients were divided into group A (23 patients, staged surgical treatment) and group B (25 patients, single-stage surgical treatment after waiting for swelling reduction) according to their soft tissue condition and preoperative informed consent status. Both group A and group B patients had visible swelling of the affected foot on admission, and some even had subcutaneous bruising and hemorrhagic blisters. However, we always recommend staged surgery for patients with severe dislocation who are determined to be at high risk of developing osteofascial compartment syndrome after a physical examination. Therefore, group A patients had a worse soft tissue condition on admission than group B patients in general. The study was approved by the ethics committee of our hospital.

### Surgical methods

All patients were immediately admitted to the hospital to perform preoperative assessments, rule out surgery contraindications, and implement appropriate treatment plans. And all the operations in this study were performed by the same group of surgeons including emergency reduction and internal fixation.

**Figure 1:**
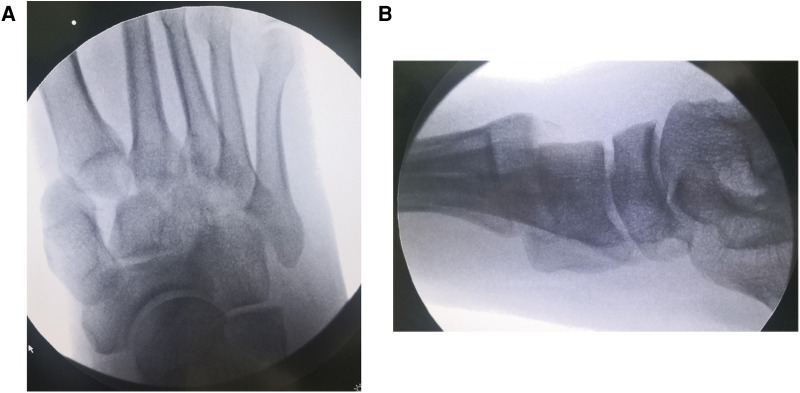
Anteroposterior and lateral radiographs of the patient's injured foot on admission, showing complete dislocation of the metatarsophalangeal joint. (**A**) Anteroposterior (**B**) Lateral.

**Figure 2:**
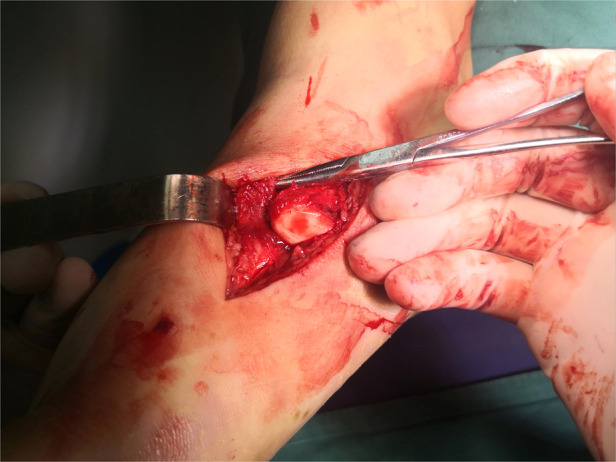
Intraoperative exploration showing the joint capsule and extensor hallucis longus tendon embedded in the first metatarsophalangeal and intercuneiform joints.

**Figure 3:**
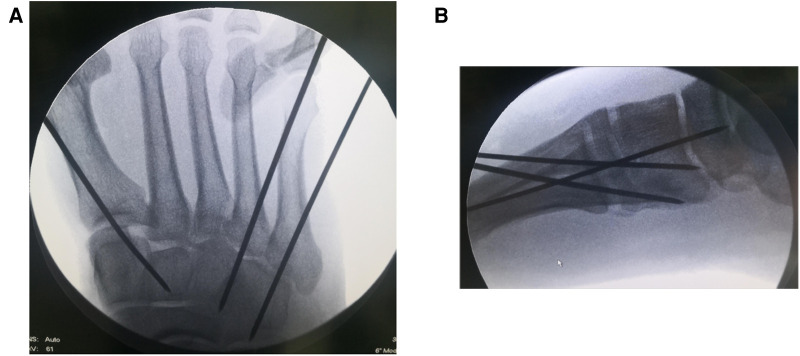
Intraoperative fluoroscopy films showing temporary fixation with Kirschner wires after satisfactory reduction. (**A**) Anteroposterior (**B**) Lateral.

All patients in group A underwent emergency stage I surgical treatment after admission. Surgery was performed in the supine position under intraspinal anesthesia or nerve blocking anesthesia with a pneumatic tourniquet. Closed reduction was first attempted under C-arm machine monitoring (Figure 1). If the reduction was satisfactory, temporary fixation of the Lisfranc joint was performed with Kirschner wires leaving only the soft tissue closure. Otherwise, a small longitudinal incision was made locally in the Lisfranc joint, to fully expose the dislocated joint, where reduction was difficult. The soft tissue and bone fragments in the joint space were then explored and quickly cleaned using a hemostat or periosteal separator under direct vision. A combination of prying and manual reduction was then performed under direct vision (Figure 2). Finally, the medial and lateral columns were temporarily fixed using Kirschner wires to restore the basic alignment (Figure 3).

**Figure 4:**
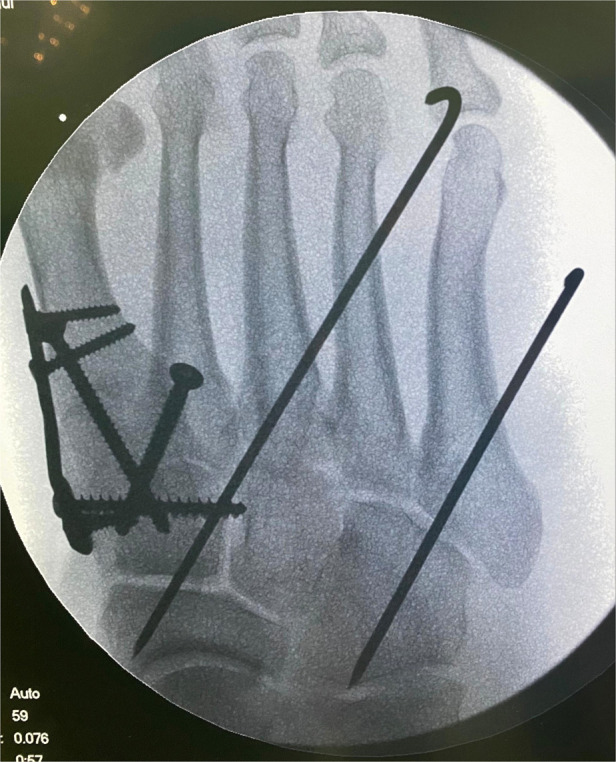
Intraoperative fluoroscopy. Stage II surgery Lisfranc injury with open reduction internal fixation and fusion of the first metatarsophalangeal joint.

Stage II surgery was performed when dermatoglyphic signs were present and there were no visible signs of local soft tissue infection or necrosis. A medial foot incision was made (if an incision and reduction were performed in stage I surgery, stage II was considered *via* the original surgical incision) to expose the injured tarsometatarsal joint. Reduction was then performed under direct vision. The first tarsometatarsal joint was fused and the tarsometatarsal joint surface was fully exposed using a spreader. Next, the articular cartilage was removed, the joint surface was freshly treated, and the first tarsometatarsal joint was fused after satisfactory reduction under direct vision. The medial cuneus was clamped to the base of the second metatarsal with a pointed reduction forceps and fixed with 3.5 mm cortical bone screws after satisfactory Lisfranc reduction. Intercuneiform joint dislocations could also be fixed laterally with 3.5 mm cortical bone screws. In cases with a shortened lateral column with cuboid bone compression fracture, a posterolateral incision was required to prop up the compressed cuboid bone and restore the length of the lateral column, followed by bone grafting and fixation of the cuboid bone fracture with a micro-steel plate. The 4th and 5th tarsometatarsal joints were then fixed with Kirschner wires (Figure 4).

**Figure 5:**
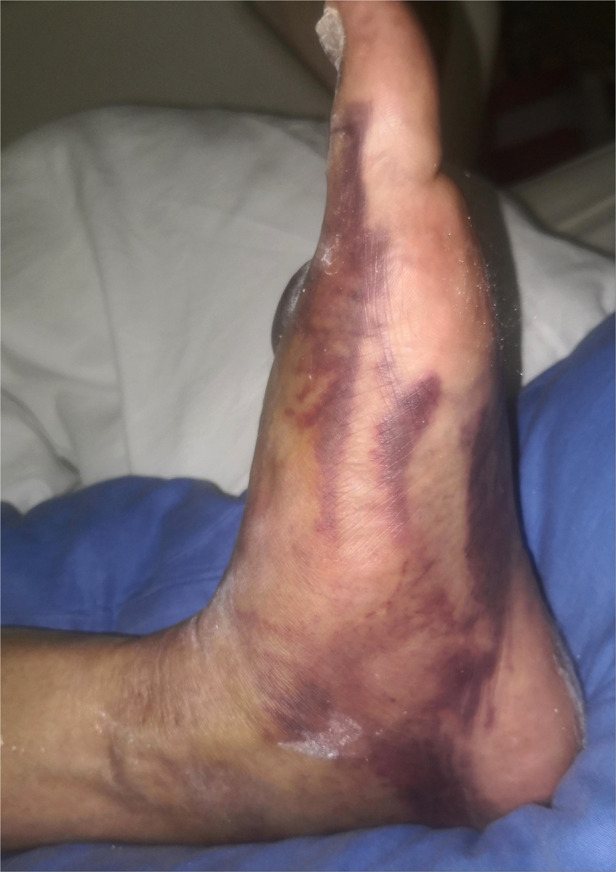
A patient admitted with severe swelling of the affected limb with dorsal foot skin blisters and extremely poor soft tissue condition.

**Figure 6:**
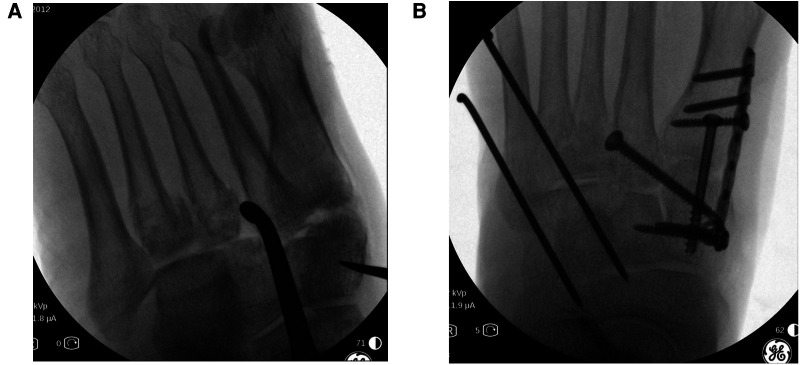
Intraoperative fluoroscopy. The patient's first metatarsophalangeal joint surface is crushed; thus, fusion surgery was performed. The dislocated fourth and fifth metatarsophalangeal joints were difficult to reduce, resulting in significantly increased operative time and intraoperative fluoroscopy. (**A**) Intraoperative C-arm machine to monitor the metatarsophalangeal joint reduction. (**B**) Postoperative lateral and anteroposterior radiographs, respectively.

All patients in group B were admitted to the hospital and treated for swelling due to the patient's extremely poor soft tissue condition (Figure 5). After the appearance of dermatoglyphic signs and ensuring that there were no visible signs of local soft tissue infection and necrosis, ORIF of Lisfranc injury were performed and the first tarsometatarsal joint was fused (Figure 6). The rest of the surgical procedure was performed in the same way as in stage II in group A.

### Postoperative management

After surgery, the affected limb was elevated to facilitate healing of the swelling, dressings were changed every other day, and the Kirschner wire holes were disinfected daily with alcohol. Passive flexion and extension exercises of the toe, ankle, and knee; straight-leg raising exercises of the quadriceps; and flexion and extension exercises of the hip and knee were started on the first day after surgery, while active flexion and extension exercises of the toe, ankle, and knee were started on the second day after surgery. The quality of fracture reduction and healing were assessed using radiographs. Discharge is based on the complete reduction of swelling in the patient's foot and the disappearance of redness and swelling around the wound, so the length of hospitalization also reflects the time required for the patient's postoperative soft tissue recovery. The stitches were removed 2 weeks after surgery. The Kirschner wire was removed 4–6 weeks after surgery and partial weight-bearing walking was permitted. Postoperative imaging was performed regularly, as well as follow-up of the functional recovery of the foot and ankle. American Orthopedic Foot and Ankle Society (AOFAS) midfoot scores and visual analog scale (VAS) scores were used for assessment at the final follow-up.

### Statistical processing

Analysis was performed using IBM SPSS Statistics for Windows, version 19.0 (IBM Corp., USA). Quantitative data were expressed as $\bar{x}\pm s$. Length of hospitalization, length of surgery, postoperative AOFAS midfoot score, and VAS score were compared between groups using paired *t*-tests. *p* < 0.05 was considered statistically significant. But power calculation was not performed for patients in this study.

## Results

**Figure 7:**
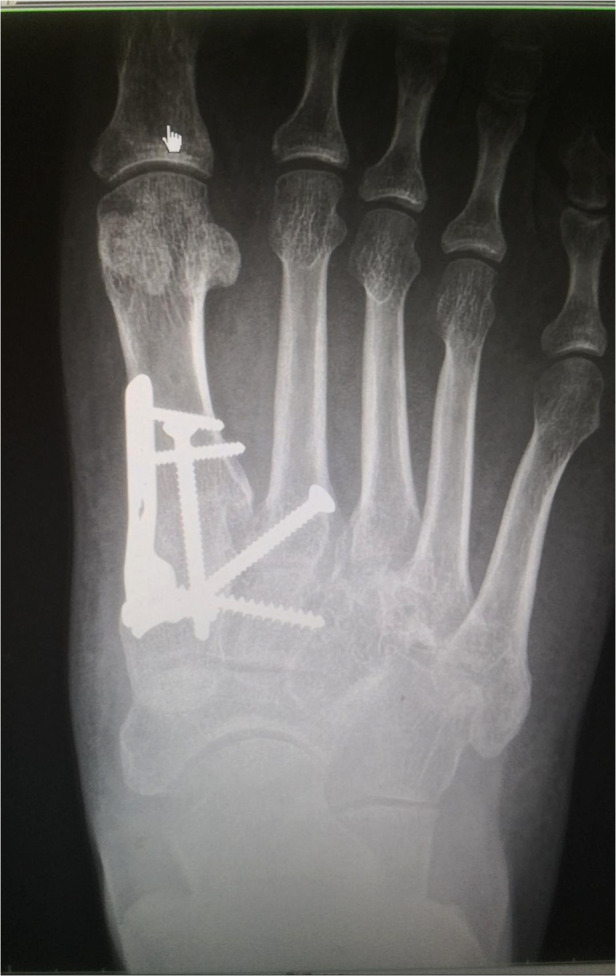
Radiographs of the foot of a patient in group A at 1 year after surgery.

A total of 48 patients with closed Lisfranc injury and severe dislocation were included, including 30 males and 18 females, 28 with right-sided injuries and 20 with left-sided injuries, and a mean age of 49.5 years (Table 1). In group A, stage I surgery was performed within 4–8 h after injury, with stage II surgery performed 6–10 day later. The length of hospitalization was 11.52 ± 1.61 day. And 19 patients in group A had a small incision to assist with repositioning during the stage I surgery. In group B, surgery was performed 10–20 day after injury and the length of hospitalization was 19.80 ± 2.37 day. The total lengths of surgery were 67.34 ± 1.71 min and 104.36 ± 8.31 min in groups A and B, respectively. No serious soft group problems were seen in either group after surgery. We used the patient's clinical presentation, complaints, AOFAS scores and radiological manifestations at the post-operative follow-up to evaluate the quality of reduction and functional rehabilitation. All 48 patients completed the final follow-up (range: 12–24 months, mean: 18 months). All fractures had healed at 12–18 weeks after surgery (mean: 14.6 weeks) according to radiographs (Figure 7). At the 1-year postoperative follow-up, the AOFAS and VAS scores during weight-bearing walking for the patients in group A were 86.87 ± 4.24 and 1.91 ± 0.78, respectively, and 71.72 ± 5.46 and 3.20 ± 1.17 in group B. The mean length of hospitalization, length of surgery, and postoperative AOFAS and VAS scores differed significantly between groups A and B (Table 2). By the end of the follow-up period, two patients in group B had developed traumatic arthritis according to the clinical symptoms of the pain during activity and radiological manifestations. No patients experienced joint re-dislocation or required secondary surgery.

**Table 1:** Demographics.

	Value
Patient (%)	48(100.0)
Male	23(48.0)
Female	25(52.0)
Age, average(range), year	49.5(18–81)
Classification of dislocation	
Homolateral, partial displacement (%)	24(50.0)
Homolateral, total displacement (%)	16(33.3)
Divergent, total displacement (%)	8(16.7)

**Table 2:** Comparisons of treatment results between patient groups.

	Group A	Group B	*p*-value
Length of hospitalization (day)	11.52 ± 1.61	19.80 ± 2.37	*p* < 0.05
Length of surgery (min)	67.34 ± 1.71	104.36 ± 8.31	*p* < 0.05
1-year Postoperative AOFAS score	86.87 ± 4.24	71.72 ± 5.46	*p* < 0.05
1-year Postoperative VAS score	1.91 ± 0.78	3.20 ± 1.17	*p* < 0.05

The mean length of hospitalization, length of surgery, and postoperative AOFAS and VAS scores differed significantly between groups A and B. Abbreviations: AOFAS, American orthopedic foot and ankle society; VAS, visual analog scale.

## Discussion

The mechanisms of the Lisfranc injuries include sprains, crush injuries, and mixed violent injuries. The high-energy mechanisms of these injuries often lead to severe dislocation and subsequent perioperative complications without proper treatment, which can negatively affect efficacy and prognosis. But how to balance the advantages and disadvantages of emergency surgery and elective surgery is still controversial. Therefore, this study investigated the best treatment option for these patients by comparing the efficacy between staged and single-stage treatment of patients with closed Lisfranc injuries and dislocation.

The staged surgical approach has been widely used in lower extremity injuries and shows good efficacy, including in Lisfranc injuries. Kadow et al. treated 123 patients with midfoot fractures or dislocations with stage I reduction and external frame fixation followed by elective ORIF surgery after the swelling subsides. They showed that staged treatment avoided incision complications related to emergency surgery. Herscovici et al. treated 176 patients with high-energy Lisfranc injuries with stage I Kirschner wire fixation and stage II ORIF. They showed that the stage I procedure was simpler with the Kirschner wire than external frame fixation and determined the reliability of the results of the reduction and fixation (11, 12). The results of this study also demonstrated significantly better length of hospitalization, length of surgery, and surgery outcomes for staged surgery compared to those of the control group. Although previous studies have emphasized the importance of staged treatment, the specific details of staged surgery have not been adequately described. For some patients, closed reduction does not achieve the desired effect. Therefore, the initial reduction can be performed with the aid of small local incisions during stage I surgery. This can also allow exploration of the extent of damage to the joint surface and prompt attention to possible tendon or vascular injuries, thus facilitating the development of a treatment plan for stage II surgery and determining patient prognosis. By making a small incision to assist in the repositioning, it not only avoids the possibility of poor closed repositioning, but also provides a certain decompression effect, resulting in a reduced probability of skin necrosis and fascial compartment syndrome, preventing further damage to nerve, vascular, and tendon structures and facilitating pain relief and swelling reduction in the affected limb. Moreover, all patients in group A had no incisional complications after stage I surgery in this study. But too many complex surgical operations should not be performed in stage I surgery under the principles of rapid reduction, reduction of medically-induced injuries, and minimization of operative time. And the location of the incision in stage I surgery should preferably also consider the stage II surgical approach to avoid multiple incisions and, thus, reduce the risk of incisional complications. For some patients in group A of this study, we used a medial incision to facilitate good reduction in the stage I surgery and this incision could be used for first tarsometatarsal joint fusion in stage II surgery. And notably, only two patients in group B had developed traumatic arthritis due to poor repositioning of the lateral column in this study. Compared to group B patients, group A patients avoided the difficulty of repositioning due to tissue adhesion, thus effectively improving the quality of reduction because of early reduction. So the quality of reduction may play an important role in the functional rehabilitation. The advantages of stage I surgery are that early emergency reduction facilitates the restoration of alignment reducing the pressure of the injury on the surrounding soft tissues which is good for swelling decongestion, and lays the foundation for stage II surgery.

However, since temporary Kirschner wires fixation often lacks sufficient strength and is prone to complications such as loosening, retraction, and wire breakage, re-operation is required after the swelling subsides to provide strong internal fixation of the intermediate and medial columns using screws, splints or more Kirschner wires. For stage II surgery, the commonly used surgical procedures are ORIF and joint fusion. However, the choice of procedures is controversial. Although there is little difference between the two procedures in terms of return to work, mobility, patient satisfaction, postoperative nerve injury infection, and other prognostic indicators, recent studies have shown the advantages of fusion surgery for the treatment of complex Lisfranc injuries, especially in patients with severe ligament and articular cartilage damage. The probability of undergoing internal fixation and secondary fusion surgery after ORIF is higher, and ligament damage cannot be fully repaired. In addition, the quality of reduction after ORIF decreases over time, leading to the development of traumatic arthritis due to joint pathology (13–18). In their evaluation of the treatment of Lisfranc injuries in young athletes, Cochran et al. reported a lower rate of endograft removal in the fusion group than in the ORIF group, as well as an earlier return to sport and higher motor test scores (19). All patients included in this study had severe dislocation; thus, all patients underwent medial column fusion, of whom a small number (two patients in group B) complained of tolerable pain during walking on follow-up, consistent with the results of the above study. Additional anatomical studies have shown that Lisfranc screws increase the stability of the Lisfranc joint and prevent diastasis of the forefoot based on medial column fusion (20–23). None of the patients in the present study experienced re-dislocation; thus, medial column fusion and Lisfranc screw fixation maintained midfoot stability, preserved the mobility of the second and third metatarsal tarsal joints, and partially preserved some functions of the metatarsal tarsal joints, thus achieving a better treatment outcome. Therefore, patients with multidirectional instability or complete dislocation of the first tarsometatarsal joint, comminuted fractures and dislocation of the first or second tarsometatarsal joint surface, and complete rupture of the Lisfranc ligament alone, fusion of the first tarsometatarsal joint may be a better choice according to the results of this study.

If the medial column is unstable during surgery, a screw or transarticular plate, or even the Kirschner wires fixation may be used. But the lateral column of the tarsometatarsal joint has a high degree of joint mobility, fusion of the lateral column can result in pain and stiffness in the midfoot. Elastic fixation of the lateral column using Kirschner wires is usually performed. However, some patients also show naviculocuneiform joint instability due to the high injury force; in these cases, the plate should span the naviculocuneiform and first tarsometatarsal joints. Therefore, the choice of internal fixation of the medial column during the second-stage surgery needs to be made according to the different conditions striving for the simplest way to achieve medial column stability.

But one must be cautions about drawing conclusions from a relatively small number of patients. More clinical studies with large samples are needed to further validate the effectiveness of this treatment in the future.

In conclusion, staged surgery to treat patients with closed Lisfranc injury with dislocation not only reduced the incidence of perioperative complications but also achieved good surgical outcomes while shortening the lengths of surgery and hospitalization, which is worthy of clinical application.

## Data Availability

The original contributions presented in the study are included in the article/Supplementary Material, further inquiries can be directed to the corresponding author/s.
